# Pericapsular nerve group block for intracapsular vs. extracapsular hip fracture

**DOI:** 10.1111/anae.70030

**Published:** 2025-10-13

**Authors:** Santi Di Pietro, Riccardo Maffeis, Laura Girón‐Arango, Eugenio Jannelli, Stefano Perlini

**Affiliations:** ^1^ University of Pavia Pavia Italy; ^2^ Toronto Western Hospital, University of Toronto Toronto ON Canada

The pericapsular nerve group (PENG) block is used increasingly for hip fracture analgesia in emergency departments [1, 2, 3, 4]. Cadaveric and clinical studies suggest that, when performed with at least 20 ml of injectate, the PENG block provides analgesia for both intracapsular and extracapsular hip fractures, although greater volumes are associated with an increased risk of undesirable spread to the femoral nerve, resulting in quadriceps weakness [2, 5, 6].

No studies have yet compared the analgesic effect of the PENG block in intracapsular vs. extracapsular hip fracture. Koh et al. investigated the analgesic effect of PENG vs. supra‐inguinal fascia iliaca block, which included a sub‐analysis of intracapsular and extracapsular groups [7]. There was no statistically significant difference in pain scores between these groups, but the study was not powered for this outcome. However, a key limitation is the inclusion of subtrochanteric fractures, for which the PENG block is not indicated [2]. This likely underestimated the analgesic effect in the extracapsular group and amplified the between‐group difference.

We conducted a similar exploratory secondary analysis of our recent trial, which investigated the analgesic effect of the PENG block vs. infra‐inguinal fascia iliaca block in the emergency department [8]. Among the 30 patients who received a PENG block with an injectate volume of 20 ml, 13 had an intracapsular fracture (subcapital or transcervical) and 17 had an extracapsular fracture (basicervical, intertrochanteric, simple pertrochanteric or multifragmentary pertrochanteric).

**Figure 1:**
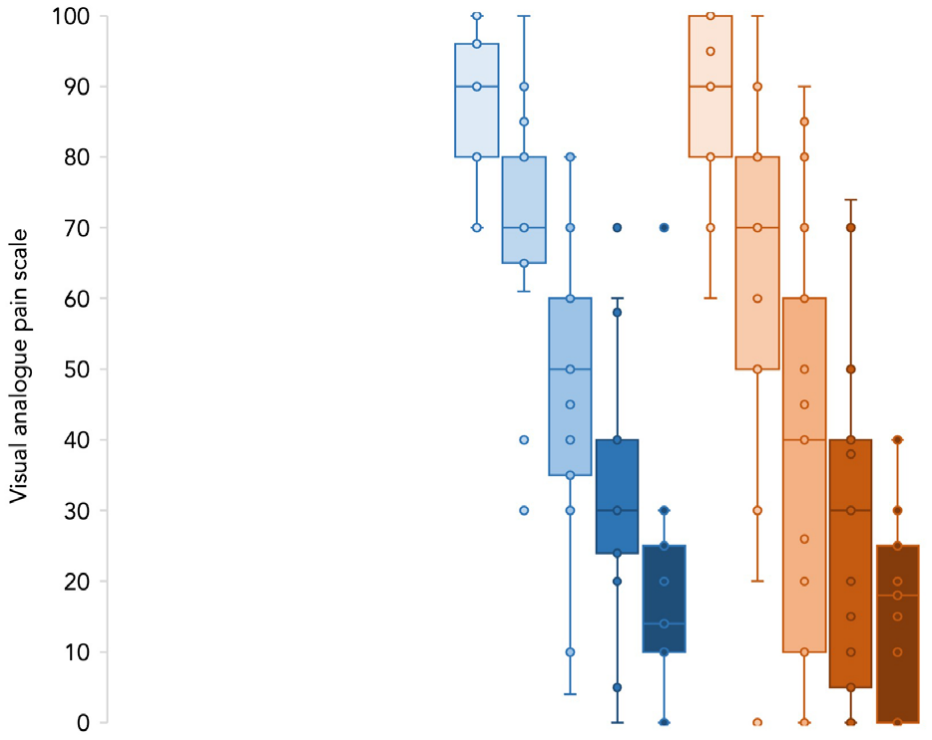
Box and whiskers plot for visual analogue pain scale values at baseline (t0), and 5 (t1), 15 (t2), 30 (t3), 60 (t4) min post block in intracapsular (blue) and extracapsular (red) fractures.

We found that the interquartile range for visual analogue pain scale values was greater for extracapsular fractures, with nearly identical Q3 values between the two groups and a lower Q1 value in the extracapsular group (Fig. 1). We calculated pain intensity differences from visual analogue pain scale values at each time‐point and the summed pain intensity differences. We then obtained the proportion of the summed pain intensity differences for the intracapsular and extracapsular groups as 68% (95%CI 53–83%) and 68% (95%CI 57–78%). These data suggest that the PENG block, performed with 20 ml injectate, may be as effective for extracapsular fractures as it is for intracapsular fractures. Nevertheless, the difference in the dispersion from median values may suggest a less consistent analgesic effect of the PENG block in extracapsular fractures, possibly due to higher variability in the injectate spread to the trochanteric area.

We present these data descriptively due to our small sample size and because our study was not powered for this secondary analysis. Future adequately powered studies should clarify if for a given injectate volume, the PENG block results in different analgesic effects in intracapsular and extracapsular fractures. It would also be worthwhile investigating whether these fracture patterns require a different injectate volume for the PENG block to be effective.
